# Sex Differences in the Outcome of Expressive Writing in Parents of Children With Leukaemia

**DOI:** 10.32872/cpe.5533

**Published:** 2022-03-31

**Authors:** Dorte Mølgaard Christiansen, Maria Luisa Martino, Ask Elklit, Maria Francesca Freda

**Affiliations:** 1 National Center of Psychotraumatology, Institute of Psychology, University of Southern Denmark, Odense, Denmark; 2 Department of Humanistic Studies, Federico II University, Naples, Italy; Philipps-University of Marburg, Marburg, Germany

**Keywords:** sex differences, gender differences, expressive writing therapy, mood states, childhood leukaemia, parental stress

## Abstract

**Background:**

Sex differences are widely reported in clinical psychology but are rarely examined in interventions.

**Method:**

This mixed-method explorative study examined sex differences in 13 mothers and 10 fathers of children in the off-therapy phase of acute lymphoblastic leukaemia. Parents underwent an expressive writing intervention using the guided written disclosure protocol (GWDP).

**Results:**

Mothers had more negative mood profiles than fathers but improved more during the intervention.

**Conclusion:**

Though preliminary, our findings highlight the importance of sex as a potential moderator of intervention and treatment outcome that could be of great clinical significance.

## Highlights

- Parents of children in remission from cancer can benefit from expressive writing.
- Expressive writing can improve mood states.
- Mothers may benefit more than fathers.
- More research on gender differences in outcomes is needed.

When a child falls severely ill, it affects the whole family and parental stress levels remain increased even after successful treatment (Hile et al., 2014). How the parents cope with the shared trauma and burden of that illness is essential to the health of the family as a whole (Morris et al., 2012). One study reported that parental stress was the strongest correlate of functional impairment in children at least 2 years following treatment for leukemia/lymphoma (Hile et al., 2014). Consequently, decreasing the stress and symptom levels of parents of children who have undergone treatment for serious illnesses is likely to benefit the whole family. However, parents receive little attention from hospital personnel and healthcare researchers beyond the first initial shock associated with the child’s diagnosis and treatment.

Inspired by the field of linguistics, Pennebaker et al. (2010) theorised that words used in a written narrative reflect the writer’s state of mind and could be used to track changes in the meaning attributed to an event (Pennebaker et al., 2010; Tausczik & Pennebaker, 2010). Building on these principles, expressive writing was designed to be used as an element in therapy or as an independent intervention to promote meaning-making and integrate traumatic content into a personal narrative (De Luca Picione et al., 2017; De Luca Picione et al., 2018; Martino & Freda, 2016; Martino et al., 2013). One such expressive writing intervention, the *Guided Written Disclosure Protocol* (GWDP) has been used to reduce distress, anxiety, and PTSD symptoms in parents of children with cancer, whereas results for depression have been less promising (Cafaro et al., 2019; Dicé et al., 2018; Duncan & Gidron, 1999; Duncan et al., 1998; Gidron et al., 2002; Martino et al., 2019; Martino et al., 2013). This protocol is designed to help participants build an increasingly complex and coherent narrative by building on themes of meaning-making, insight, emotion-regulation, mastery and self-efficacy (Baikie & Wilhelm, 2005).

One factor that likely affects how parents respond to different interventions is sex, but research remains scarce (Christiansen, 2015, 2017; Ogrodniczuk, 2006). Research on sex differences in the outcomes of different interventions is often limited by small sample sizes (especially few male participants), yet effect sizes are rarely reported. Furthermore, moderation effects are rarely based on a priori hypotheses. Possibly as a consequence of poor statistical power, few studies have reported significant sex differences. Nonetheless, there are indications that women generally benefit more from psychotherapy than men (Christiansen, 2015, 2017; Ogrodniczuk, 2006; Wade et al., 2016), especially interventions focusing on verbal processing of traumatic content (Christiansen, 2017). Little focus has been given to potential sex differences in outcome of expressive writing. One meta-analysis found that percentage of male participants was positively associated with effect size across 13 studies. However, this effect was not found for psychological outcomes. Furthermore, as trauma type was not controlled, it may be that women were more likely to write about more toxic exposures, such as sexual trauma, which is more common among women (Christiansen, 2017). Other studies have generally failed to report sex differences in the effects of expressive writing (Pennebaker & Chung, 2011), though this may be at least partly due to low statistical power. To the best of our knowledge, no studies have examined whether references to emotion and cognition predict treatment outcome in both men and women.

Knowledge on sex differences in how parents respond to different interventions may help improve outcomes for both individual parents and their family, not least the children whose functioning is often very dependent on the psychological health of their parents (Morris et al., 2012). In the present study we examined how a brief intervention of expressive writing affects the mood states of parents whose children were in the off-therapy ALL phase (i.e. remission of malignant cells; interruption of radio/chemotherapy; ca. 2 years post diagnosis). We chose to focus on this phase because parents whose children were not in remission would likely be too focused on the current threat to their child to fully benefit from the intervention, yet this phase remains an extremely vulnerable period within which families begin to return to “normal” life, yet parents still feel vulnerable and may need help processing the trauma (Martino et al., 2013). The present study was a pilot study implementing an expressive writing protocol in a group of parents in a very sensitive period following a serious threat to their children. The purpose was to examine sex as a moderator of the impact of expressive writing on mood states over time. We expected mothers to benefit more from the intervention than fathers.

## Method

### Participants

Participants included 10 fathers and 13 mothers whose children were at the beginning of the off-therapy remission phase being treated for acute lymphoblastic leukemia at one of Italy’s leading facilities for children with neoplastic illness. The mean age was 41.5 years (*SD* = 5.01) for fathers and 38.2 years (*SD* = 5.6) for mothers. The children undergoing treatment were four boys (*M* = 4.25 years, *SD* = 0.5) and nine girls (*M* = 6.77 years, *SD* = 3.3).

### Procedure

The sample was consecutive with parents being identified from medical reports. Recruitment occurred through phone calls or at the hospital. Parents were contacted one day after their child was confirmed to be in remission. Exclusion criteria were ongoing therapy/interventions for symptoms related to dealing with their child’s illness. Participation was voluntary and confidential based on informed written consent, and the study was approved by the hospital’s ethics committee.

The GWDP protocol was used in the present studies because of the above mentioned positive results in parents of children with cancer. Writing sessions lasted 30 minutes and were conducted individually in a quiet room of the hospital with only the psychologist researcher present. In the first session parents were asked to describe events as they occurred and developed over time. In the second session (10-15 days later) parents were invited to express the emotions accompanying these same events. In the final session (10-15 days later) parents were instructed to envision their future, compare their present and past feelings, consider the effects the experience has had on them, and describe how they expect to cope with future adversities. Following the intervention parents were assessed for need of continued psychological support. One mother was offered and accepted additional meetings with a psychologist at the hospital.

The study originally included a control group of 23 parents not undergoing a writing intervention who were invited to participate during the subsequent year. However, as the two groups differed significantly on the main outcome measure at T_1_, prior to intervention, we unfortunately had to exclude the control group, as it would be impossible to conclude whether any potential differences between the groups were caused by the intervention or by other factors. Out of a total of 20 couples whose children were diagnosed during 2007, seven couples and an additional three fathers declined participation, thus leaving us with 10 parental dyads and three mothers without participating partners. Participants were assessed prior to the intervention (T_1_), 10-15 days post-intervention (T_2_) and at follow-up (40-45 days post-intervention (T_3_).

### Measure

The Profile of Mood States (POMS) is a self-report questionnaire assessing specific affective states during the past week (McNair et al., 1971). The test consists of 58 adjectives belonging to six factors: tension–anxiety, depression–dejection, anger–hostility, vigor–activity, fatigue–inertia, and confusion–bewilderment. Items are rated on a 5-point Likert scale ranging from 0 (not at all) to 4 (extremely). The POMS scale revealed acceptable reliability across all three measurements (Cronbach’s α > .96) and across five of the six subscales (Cronbach’s α > .81). Cronbach’s α was consistently low for the vigor-activity subscale and an inter-item correlation matrix revealed internal inconsistencies. Thus, this subscale was excluded from all analyses. A measure of change in POMS scores was calculated for later analyses (T3 scores – T1 scores) with negative scores indicating an improvement in mood states.

### Data Analyses

The low number of participants (*N* = 23) limited the type and power of statistical analyses. Therefore, the results must be considered preliminary. A significance level of *p* < .05 was used but to better guide future research, high and medium effect sizes are also reported regardless of statistical significance level. A *t*-test was also used to examine sex differences in changes in POMS total and subscale scores between assessments. Effect sizes were calculated using Cohen’s *d* with values of 0.2, 0.5, and 0.8 used as guidelines for small, medium, and large effect sizes, respectively. The main effect of time was examined along with the main effect of sex and the interaction effect between time and sex in a mixed methods within-between subjects ANOVA. Effect sizes were calculated using partial Eta squared (ηp^2^) with values of .01, .06, and .14 used as guidelines for small, medium, and large effect sizes, respectively. Due to the way in which data was collected and stored, it was not possible to conduct paired analysis based on parental dyads. Thus, analyses of sex differences fail to take into account that paired parents affect each other and share both the child and the circumstances surrounding that child’s illness and treatment.

## Results

The mixed methods ANOVA for mothers and fathers across all three measurements is shown in Table 1a, 1b, and 1c. In accordance with the *t*-tests, significant large main effects of sex were found for POMS total score, *F*(1, 21) = 7.77; *p* < .05; $\eta_{p}^{2}$ = .27, and all subscale scores, $\eta_{p}^{2}$ > .20; *p* < .05), except for depression-dejection (*p* = .07; $\eta_{p}^{2}$ = .14; see Table 1a). No significant main effect was found for time on POMS total score despite a relatively large effect size ($\eta_{p}^{2}$ = .17; see Table 1b). There was, however, a significant main effect on tension-anxiety (Wilk’s lambda = .70, *F*(2, 20) = 4.26, *p* < .05, $\eta_{p}^{2}$ = .30). The main effects for time on the remaining subscales were all medium though non-significant (.08 < $\eta_{p}^{2}$ < .20). All effect sizes indicated a decrease in POMS levels from pre-treatment to follow-up. Finally, the interaction effects between sex and time were all non-significant, though all except for the depression-dejection subscale had effect sizes that can be considered medium-to-large (.06 < $\eta_{p}^{2}$ < .17; please see Table 1c).

**Table 1a:** ANOVA: Between-Subjects Effect – SEX

POMS subscale	*F*	*p*	*PE* ^2^
Total	7.77	< .05	.270
Tension-anxiety	7.52	< .05	.264
Depression-dejection	3.63	.07	.147
Anger-hostility	5.45	< .05	.206
Fatigue-inertia	8.90	< .01	.298
Confusion-bewilderment	21.10	< .001	.501

*Note.* POMS: Profile of Mood States.

**Table 1b:** ANOVA: Multivariate Tests – Time

POMS subscale	Wilk’s λ	*F*	*p*	*PE* ^2^
Total	.828	2.07	ns.	.172
Tension-anxiety	.701	4.26	< .05	.299
Depression-dejection	.915	0.93	ns.	.085
Anger-hostility	.900	1.12	ns.	.100
Fatigue-inertia	.806	2.41	ns.	.194
Confusion-bewilderment	.912	0.96	ns.	.088

*Note.* POMS: Profile of Mood States.

**Table 1c:** ANOVA: Multivariate Tests – Time * SEX

POMS subscale	Wilk’s λ	*F*	*p*	*PE* ^2^
Total	.887	1.28	ns.	.113
Tension-anxiety	.835	1.97	ns.	.165
Depression-dejection	.960	0.41	ns.	.040
Anger-hostility	.898	1.14	ns.	.102
Fatigue-inertia	.869	1.50	ns.	.131
Confusion-bewilderment	.934	0.71	ns.	.066

*Note.* POMS: Profile of Mood States.

Post-hoc *t*-tests examining the moderation effects (please see Table 2) revealed that mothers reported a higher average decrease in POMS scores (*M* = -20.46, *SD* = 54.43) compared to fathers (*M* = -6.20, *SD* = 8.04) from T_1_ to T_3_, though the effect size was small and non-significant (Cohen’s *d* = .37). The main decrease in POMS levels in mothers occurred during the intervention (*M* = -21.54) with little additional change occurring afterwards (*M* = -1.08; see Table 2). This difference was much smaller in fathers (*M* = -4.7 vs. *M* = -1.5). Though independent t-tests examining sex differences in the decrease in POMS scores at each step were non-significant, a large effect size was found comparing the decrease in POMS scores during the intervention (*d* = .54) but not subsequently (*d* = .23). These sex differences were not significant for any of the subscales and could only be considered medium for the tension-anxiety subscale (*d* = 0.70). Most participants (78%) experienced some decrease in their POMS scores over the course of writing. However, 10.0% of fathers and 30.8% of mothers reported some increase in POMS scores from T_1_ to T_3_.

**Table 2:** POMS Total and Subscale Scores at Baseline and Over Time

POMS total score	*M* (*SD*)			*t*	*p*	*d*
	All	*F*	*M*			
POMS total scores						
POMS total T_1_	52.61 (39.15)	27.80 (25.02)	71.69 (37.86)	3.16	< .05	1.37
POMS total T_2_	38.39 (34.41)	23.10 (22.43)	50.15 (38.06)	1.99	.060	0.87
POMS total T_3_	38.35 (38.64)	21.60 (20.06)	51.23 (44.97)	2.12	< .05	0.85
Change from T_1_ to T_3_						
POMS total	-14.26 (41.17)	-6.20 (8.04)	-20.46 (54.43)	0.93	ns.	0.37
Tension-anxiety	-4.91 (8.05)	-2.00 (3.56)	-7.15 (9.84)	1.75	ns.	0.70
Depression-dejection	-3.52 (14.21)	-1.90 (1.79)	-4.77 (19.08)	0.54	ns.	0.21
Anger-hostility	-2.22 (9.26)	-1.00 (2.05)	-3.15 (12.32)	0.62	ns.	0.24
Fatigue-inertia	-2.43 (6.06)	-1.40 (1.35)	-3.23 (8.02)	0.81	ns.	0.32
Confusion-bewilderment	-1.17 (5.16)	0.10 (1.29)	-2.15 (6.72)	1.18	ns.	0.47

*Note.* F = fathers; M = mothers. *t*, *p*, and Cohen’s *d* all relate to sex differences. *POMS* = Profile of Mood State. T_1_: prior to therapy; T_2_: at the end of therapy: T_3_: at follow-up.

## Discussion

As the present study was a pilot study, the most relevant finding was that the implementation of the emotional writing procedure in this clinical setting was successful. That significant sex differences were found despite low statistical power highlights the importance of taking sex into account in intervention studies. Several of the non-significant effects could be considered moderate or even strong, indicating that type II error due to small sample size may have disguised further significant findings. Mothers reported significantly more negative mood states than fathers. It is possible that mothers were more negatively affected by their child’s illness, as indicated by prior research (Christiansen, 2017; Clarke et al., 2009). Another possibility is that these findings may just reflect sex differences in everyday mood.

Although there was no significant main effect of time, the effect size was quite large for POMS total scores, and medium-to-large effect sizes were found for all five POMS subscales. Without a control group, it is unknown whether the apparent improvements in mood profiles were caused by the intervention or simply by the passing of time. However, mothers showed a much steeper decline in symptom scores from T_1_ to T_2_ than from T_2_ to T_3_. This decline was larger in mothers than in fathers during the intervention, whereas there was no difference between the two sexes in changes in mood states occurring from the end of the intervention until follow-up. This may suggest that, at least in mothers, some of the changes were caused by the writing intervention. Accordingly, although the interaction between time and sex was statistically non-significant, the medium-to-large effect sizes found for both POMS total score and all but one of the subscale scores suggests that future studies may reveal women to benefit more from expressive writing interventions than men. Though preliminary, our findings highlight the importance of including sex as a moderator in treatment studies.

Though not shown here, analyses using the Linguistic Inquiry and Word Count software (LIWC) (Pennebaker et al., 2007; Freitag et al., 2011; Freda & Martino, 2015) found that mothers focused more on affect during writing sessions than fathers, including both positive and negative emotions (analyses may be obtained from corresponding author). This is in accordance with findings from prior studies (Newman et al., 2008; Thomson & Murachver, 2001). Ogrodniczuk (2006) suggested that women’s willingness to self-disclose and express emotion make them better patients and help them benefit more from therapy. Perhaps the socialization processes that cause women to share emotional content with others more easily than men make them more prepared to benefit from interventions focusing on emotional processing. Another possibility is that women’s stronger inclination to seek treatment (Christiansen, 2015; Ogrodniczuk, 2006) makes them generally more prepared than men to put in the effort needed for it to be successful. Though the present study was not based on a treatment-seeking sample, mothers were more likely than fathers to agree to participate, so a similar phenomenon may be present in this sample. Whereas it is possible that mothers’s scores simply declined more because they were higher from the beginning, thus leaving more room for improvement, this would also be the case from the end of treatment to follow-up where no additional change occurred. This may suggest that the decrease was in fact caused by the writing intervention, but due to the unfortunate exclusion of the control group, there is no way of knowing for sure.

Finally, it is important to note that four of the mothers and one father experienced an increase in symptoms over the course of the writing intervention. Thus, whereas mothers on average benefitted more, they also appear more likely to get worse over time. As the POMS measure was not directly linked to the child’s illness and only assessed mood states during the past weeks, the increased POMS scores may have been caused by new chronic or temporary stressors, independently of the intervention. However, such findings does serve as a reminder that when evaluating the benefits of any intervention, we must focus both on overall gains and on potential detrimental individual effects.

Sex differences in intervention outcomes is of great importance to both scientists and clinicians, and implementing these into treatment and intervention designs may increase the benefits of these for both men and women presenting with a variety of symptoms, thus reducing the great societal and personal costs associated with ineffective interventions (Christiansen, 2017; Donner & Lowry, 2013). Knowledge about both sex and gender and how they influence intervention outcomes should be implemented in research on different types of psychotherapy to a much greater degree than what is currently being done (Bekker & van Mens-Verhulst, 2007; Christiansen, 2015; Christiansen & Berke, 2020; Christiansen & Elklit, 2012). Whereas the results of the present study are preliminary and cannot in and of themselves be used as evidence of sex differences in the therapeutic effects of expressive writing and emotional processing in general, it is our great hope that it may increase focus on the importance of considering sex differences in the impact of psychotherapy and psychological interventions. In terms of clinical implications, taking sex differences into account when designing and selecting interventions for parents of critically ill children may help reduce symptom levels for both mothers and fathers and in turn improve quality of life for the whole family.

### Strengths and Limitations

Beyond showing the feasibility of such an intervention in a sensible clinical environment, the primary strength of this study is the specific focus on sex as a potential moderator of intervention outcome. However, the fact that the study was not originally designed with this in mind, thus failing to ensure sufficient power for detecting significant effects, severely limits the conclusions. The exclusion of the control group due to significant pre-treatment differences in POMS scores severely limits the results, as we were not able to conclude whether the reductions in POMS scores over time were in fact caused by the intervention. Further, low sample size of this pilot trial only allows cautious interpretation of results. Finally, the inability to match mothers and fathers into parental dyads forced us to treat the two sexes as independent groups, thereby making our results vulnerable to certain biases, such as parents affecting the symptom levels of their partners and both parents being affected by how their child copes with the illness along with other shared circumstances and stressors.
